# Amotio-retinae-Versorgung während der Corona-Pandemie: Suchten Patienten mit Netzhautablösung während der Corona-Pandemie später den Augenarzt auf? Ergebnisse aus einer deutschen Universitätsaugenklinik

**DOI:** 10.1007/s00347-020-01248-6

**Published:** 2020-10-10

**Authors:** N. Kaupke, M. S. Spitzer, R. Kromer

**Affiliations:** Klinik und Poliklinik für Augenheilkunde, Universitätsklinikum Hamburg-Eppendorf (UKE), Martinistr. 52, 20246 Hamburg, Deutschland

**Keywords:** Netzhautablösung, COVID-19, SARS-CoV‑2, Deutschland, Ophthalmologischer Notfall, Retinal detachment, SARS-CoV‑2, COVID-19, Ophthalmological emergency, Germany

## Abstract

**Hintergrund:**

Eine Amotio retinae ist ein ophthalmologischer Notfall. Verzögerung der Diagnose oder Therapie kann die Visusprognose reduzieren. Aus Presseberichten geht hervor, dass Notfallpatienten aus Angst vor einer Ansteckung mit dem Coronavirus SARS-CoV‑2 erst deutlich verspätet einen Arzt aufsuchen und die Diagnose und Therapie verschleppen. Diese Arbeit untersucht, ob die Corona-Pandemie Auswirkungen auf die Versorgung von Patienten mit Netzhautablösung hatte.

**Methodik:**

Für diese Studie wurden 60 Patienten mit im Zeitraum 15.03.–05.05.2020 in der Augenklinik des Universitätsklinikum Hamburg-Eppendorf operativ versorgter rhegmatogener Netzhautablösung retrospektiv eingeschlossen. Als Vergleichszeitraum wurde der entsprechende Vorjahreszeitraum gewählt. Signifikante Unterschiede zwischen den Vergleichsgruppen wurden mittels Hypothesentests untersucht.

**Ergebnisse:**

Im Vergleich zum Vorjahreszeitraum zeigten sich während der Corona-Pandemie keine signifikanten Unterschiede von Geschlecht, Alter, Symptomdauer, vorherigem Arztbesuch, präoperativem Visus, Makulabeteiligung, Amotioausdehnung, Vorhandensein von proliferativer vitreoretinaler Retinopathie (PVR), Operationsverfahren oder Operationsdauer. Während der Corona-Pandemie gaben 29 % der befragten Patienten mit Amotio retinae generelle Sorgen um ihre Gesundheit aufgrund des Coronavirus SARS-CoV‑2 an.

**Diskussion:**

Während der Corona-Pandemie zeigte sich eine unveränderte Versorgung von Patienten mit Amotio retinae. Im Gegensatz zu anderen medizinischen Notfällen zeigte sich keine reduzierte Fallzahl oder erhöhte Morbidität im Vergleich zum Vorjahreszeitraum.

## Abstract (en)

**Background:**

Retinal detachment is an ophthalmological emergency. Delayed diagnostics and treatment increase the risk of permanent loss of vision. Current media reports have suggested that patients with medical emergencies delay seeking treatment out of fear of being infected with the corona virus SARS-CoV‑2. This study analyzed data from a German university hospital to determine if the coronavirus pandemic had an impact on treatment and visual outcomes of patients with retinal detachment.

**Methods:**

In this study 60 patients treated for rhegmatogenous retinal detachment in the eye hospital of the University Hospital Hamburg-Eppendorf between 15 March and 5 May 2020 were retrospectively analyzed. Patients from the corresponding period of the previous year acted as a control group. Significant differences between the groups were investigated by hypothesis testing.

**Results:**

When compared to the period in the previous year there were no significant differences for sex, age, eye, length of symptoms, previous visit to doctor, visual acuity, macula status, degree of retinal detachment, proliferative vitreoretinopathy and type or length of procedure during the coronavirus pandemic. Of the patients with retinal detachment 29% had general health concerns due to the coronavirus pandemic.

**Conclusion:**

Medical treatment for retinal detachment was not influenced by the coronavirus pandemic. In contrast to other medical emergencies the morbidity of retinal detachment did not increase and admitted cases did not decrease during the pandemic when compared to the same period in the previous year.

Eine Netzhautablösung stellt einen ophthalmologischen Notfall dar. Eine Verzögerung der Diagnose oder Therapie kann die Visusprognose reduzieren. Aus aktuellen Presseberichten geht hervor, dass Notfallpatienten beispielsweise mit Myokardinfarkt oder Apoplex aus Angst vor einer Ansteckung mit dem Coronavirus erst deutlich verspätet einen Arzt aufsuchen und somit die Diagnose und Therapie verschleppen. Die vorliegende Arbeit untersucht, ob die Corona-Pandemie Auswirkung auf die Versorgung und den präoperativen Visus von Patienten mit Netzhautablösung hatte.

Eine Amotio retinae ist ein ophthalmologischer Notfall und führt unbehandelt zu einem progredienten Visusverlust [7, 10, 16, 21, 24]. Die häufigste Form ist dabei eine rhegmatogene Amotio retinae. Ziel der primär operativen Therapie ist das Wiederanlegen der Netzhaut [24]. Ein anatomischer Erfolg wird dabei in 85–90 % der Operationen erreicht [9, 16, 17, 21]. Der funktionelle Erfolg ist v. a. abhängig von einer Mitbeteiligung der Makula [6, 13]. Das Risiko einer Makulabeteiligung steigt dabei mit zunehmender Dauer der Netzhautablösung signifikant an [1, 4, 9].

In der Regel wird eine Netzhautablösung nicht im Rahmen einer augenärztlichen Routineuntersuchung, sondern durch den Patienten selbst bemerkt [7, 11]. Zwar führen auch verspätete ärztliche Diagnosen und organisatorische Abläufe zu einer verzögerten Therapie, die anteilig größte Verzögerung entsteht jedoch durch ein verspätetes Aufsuchen des Arztes durch den Patienten [11]. Somit kommt dem Patienten eine große Verantwortung für eine zeitnahe Behandlung zu.

Die jährliche Inzidenz einer Amotio retinae beträgt in Europa etwa 1 Fall pro 10.000 Einwohner [15, 24]. Damit betrifft diese Erkrankung jährlich etwa 8300 Patienten in Deutschland und ist einer der häufigsten ophthalmologischen Notfälle mit akuter Gefahr für die Sehkraft [7, 25]. Das Risiko einer Netzhautablösung steigt mit zunehmenden Alter, insbesondere ab dem 50. Lebensjahr [18, 19]. Damit betrifft dieses Erkrankungsbild v. a. das Patientenkollektiv, das auch ein deutlich erhöhtes Risiko für einen schweren Verlauf einer COVID-19-Infektion hat [14, 26].

Aus aktuellen Presseberichten geht hervor, dass Notfallpatienten z. B. mit Myokardinfarkt oder Apoplex aus Angst vor einer Ansteckung mit dem Coronavirus SARS-CoV‑2 erst deutlich verspätet einen Arzt aufsuchen und somit Diagnose und Therapie verschleppen [8, 12]. Diese Arbeit soll mittels retrospektiver Analyse von operativ versorgten Netzhautablösungen einer deutschen Universitätsklinik untersuchen, ob sich Patienten mit Netzhautablösung während der Corona-Pandemie verspätet beim Augenarzt vorstellen und ob dies eine Auswirkung auf die Schwere der Amotio retinae hat.

## Material und Methoden

Für diese Studie wurden alle Patienten mit einer im Zeitraum 15.03.–05.05.2020 in der Augenklinik des Universitätsklinikum Hamburg-Eppendorf operativ versorgten rhegmatogenen Amotio retinae eingeschlossen (*n* = 60, mittleres Alter: 57,5 ± 14,6 Jahre). Als Studienbeginn wurde das Datum des Inkrafttretens einer weitreichenden Allgemeinverfügung gewählt, die zum Schutz vor der Corona-Pandemie das öffentliche Leben in Hamburg deutlich einschränkte [3]. Der Studienzeitraum endete mit der Gültigkeit der Allgemeinverfügung und fiel mit der schrittweisen Lockerung der bundesweiten Ausgangsbeschränkungen zusammen [2]. Als Vergleichsgruppe wurden Patienten aus dem entsprechenden Vorjahreszeitraum eingeschlossen (*n* = 48, mittleres Alter: 62,5 ± 12,3 Jahre). Von der Studie ausgeschlossen wurden Fälle mit erneuter Netzhautablösung am gleichen Auge sowie während eines stationären Aufenthaltes aufgetretenen Netzhautablösungen. Die Datenerhebung erfolgte retrospektiv und anonymisiert. Neben Alter, Geschlecht und betroffenem Auge wurde der Visus als bester korrigierter Visus bei Vorstellung im Krankenhaus erfasst. Eine Beurteilung der Makulabeteiligung erfolgte mittels optischer Kohärenztomographie (OCT). War eine OCT-Untersuchung nicht möglich, wurde der Makulastatus ebenso wie das Ausmaß der Amotio retinae anhand des fundoskopischen Befunds und ggf. weiterer apparativer Diagnostik (z. B. Weitwinkelfundusfotografie, Echographie) erhoben. Aus den Operationsberichten wurden das Vorhandensein von proliferativer Vitreoretinopathie, das Operationsverfahren (Pars-plana-Vitrektomie mit Silikonöl oder Gas, Plombenoperation mit Kryoretinopexie) sowie die Operationsdauer erhoben.

In der klinischen Routine wurden ohne standardisierten Fragebogen weiterhin erfragt und ausgewertet:

1. Anamnestische Symptomdauer in Tagen bis zur Vorstellung in der Klinik.
2. Erfolgte bereits ein ambulanter Augenarztbesuch aufgrund der Symptome?
3. Erfolgte die Vorstellung in der Augenklinik aufgrund der Corona-Pandemie verzögert? (Zum Beispiel: Konkrete Verzögerung aufgrund geänderter Öffnungszeiten des Augenarztes? Verzögerung aus Angst vor Ansteckung? Bestehen allgemeine Gesundheitssorgen aufgrund der Pandemie?)

Die statistische Auswertung erfolgte mittels SPSS 25 (IBM, New York, USA). Signifikante Gruppenunterschiede wurden mittels Hypothesentests identifiziert. Die Normalverteilung von ordinalskalierten Daten wurde mittels D’Agostino & Pearson-Test geprüft. Falls keine Normalverteilung vorlag, wurde der Mann-Whitney-Test verwendet, sonst der ungepaarte t‑Test. Kategoriale Variablen wurden mittels des einfachen Chi-Quadrat-Tests mit Yates-Korrektur bzw. bei mehr als 2 Variablen mittels des Chi-Quadrat-Tests verglichen. Es wurde ein Signifikanzniveau von *p* = 0,05 gewählt. Um eine statistische Auswertung zu ermöglichen, erfolgte eine Transformation der Visuswerte in logMAR [22]. Dabei entspricht beispielsweise der Visus von 1,0 im Dezimalsystem einem LogMAR von 0 und ein Dezimalvisus von 0,1 einem LogMAR von 1.

## Ergebnisse

Im 7‑wöchigen Studienzeitraum (15.03.–05.05.2020) wurden während der Corona-Pandemie im Universitätsklinikum Hamburg-Eppendorf 60 Patienten mit rhegmatogener Amotio retinae operativ versorgt. Im gleich langen Vorjahreszeitraum (2019) waren es 48. Im Vergleich zum Vorjahreszeitraum zeigten sich keine signifikanten Unterschiede der Patientencharakteristika Geschlecht, Alter, betroffenes Auges, Symptomdauer, vorheriger Arztbesuch, präoperativer Visus, Makulabeteiligung, Amotioausdehnung, Vorhandensein von proliferativer vitreoretinaler Retinopathie (PVR), Operationsverfahren und Operationsdauer (Tab. 1).

**Table 1:** 

	Corona-Pandemie 2020 *n* = 60	Kontrollzeitraum 2019 *n* = 48	*p*-Wert
Geschlecht	30 % weiblich	33 % weiblich	0,2020^a^
Alter bei Operation (Jahre)	57,5 ± 14,6	62,5 ± 12,3	0,8712^b^
Betroffenes Auge	45 % rechtes Auge	44 % rechtes Auge	0,8966^b^
Symptombeginn (Tage bis zur Operation)	7,0 ± 8,4	7,6 ± 11,8	0,6040^a^
Vorhergehender Augenarztbesuch	54 % ja (insgesamt *n* = 46)	49 % ja (insgesamt *n* = 43)	0,7584^b^
Visus präoperativ (LogMAR)	1,1 ± 0,8	1,0 ± 0,9	0,4155^a^
Makulabeteiligung	57 % ja	58 % ja	0,8618^b^
Betroffene Uhrzeiten (h)	6,4 ± 2,3	6,4 ± 2,3	0,9019^a^
PVR	22 % ja	19 % ja	0,8938^b^
Operationsverfahren	48 % ppV mit Gas 47 % ppV mit Öl 5 % Plombe	67 % ppV mit Gas 31 % ppV mit Öl 2 % Plombe	0,1502^c^
Operationsdauer (min)	43 ± 15,4	39 ± 10,5	0,2117^a^

^a^Mann-Whitney-Test

^b^Einfacher Chi-Quadrat-Test mit Yates-Korrektur

^c^Chi-Quadrat-Test

*PVR* proliferative vitreoretinale Retinopathie, *ppV* Pars-plana-Vitrektomie

Während der Corona-Pandemie gaben 29 % der diesbezüglich befragten Patienten (*n* = 35) an, sie hätten aufgrund des Corona-Virus grundsätzlich Sorge um ihre Gesundheit. Der einzige Patient, der eine verzögerte ärztliche Vorstellung aus Sorge vor einer Ansteckung mit dem COVID-19-Virus angab, bemerkte erst seit 1 Tag Symptome. Bei Vorstellung in der Augenklinik gaben Patienten ohne Gesundheitssorgen aufgrund der Corona-Pandemie (*n* = 25) eine Symptomdauer von 6,4 ± 6,9 Tagen und einen präoperativen Visus (LogMAR) von 1,1 ± 0,8 an. Patienten mit pandemiebedingten Gesundheitssorgen (*n* = 10) zeigten eine Symptomdauer von 4,1 ± 3,3 Tagen und einen präoperativen Visus (LogMAR) von 1,0 ± 0,9. Die Unterschiede der Symptomdauer (*p* = 0,5) und des präoperativen Visus (*p* = 0,44) zeigten sich statistisch nicht signifikant (Mann-Whitney-U-Test).

## Diskussion

Wir konnten zeigen, dass Patienten mit rhegmatogener Amotio retinae während der Corona-Pandemie im Vergleich zum Vorjahreszeitraum weder ein signifikant längeres Symptomintervall bis zur Vorstellung in der Augenklinik noch einen schlechteren präoperativen Visus aufwiesen. Während der Corona-Pandemie zeigten sich für Patienten mit bzw. ohne Gesundheitssorgen aufgrund der Corona-Pandemie keine signifikanten Unterschiede von Symptomdauer und präoperativem Visus.

Die durchschnittliche Symptomdauer bis zur Vorstellung in der Klink war in der vorliegenden Untersuchung geringer als in der Literatur beschrieben [11, 20, 21]. Ein Vergleich mit Gesundheitssystemen, in denen der Hausarzt als Gatekeeper vor einem Facharztbesuch fungiert, ist jedoch nur bedingt aussagekräftig.

Mit Ausnahme einer größeren Patientenanzahl zeigten sich keine statistisch relevanten Unterschiede zum Vorjahreszeitraum. Eine tatsächliche Erhöhung der Fallzahlen kann in dieser Arbeit nicht ausgeschlossen werden. Das erhöhte Patientenaufkommen in der Studienklinik ist jedoch wahrscheinlich auf reduzierte Kapazitäten der umliegenden Kliniken im Rahmen von Coronavirus-Beschränkungen zurückzuführen.

Den Autoren ist keine weitere Untersuchung der Auswirkungen der Corona-Pandemie auf ophthalmologische Notfälle bekannt. Untersuchungen anderer medizinischer Notfälle aus den USA und Italien zeigten während der Corona-Pandemie jedoch eine annähernde Halbierung der Hospitalisierungsraten für Myokardinfarkte bei erhöhter Mortalität und Morbidität der Fälle [5, 23]. Warum sich das Patientenverhalten zwischen verschiedenen medizinischen Notfällen unterscheidet, lässt sich nur mutmaßen. Zum einen waren die USA und Italien von der Corona-Pandemie deutlich schwerer betroffen, sodass medizinische Kapazitäten ggf. eingeschränkter und die Sorge vor einer Ansteckung als Hindernis für ein Aufsuchen des Krankenhauses möglicherweise größer waren als in Deutschland. Zum anderen zeigten in unserer Arbeit Patienten trotz genereller Gesundheitssorgen aufgrund der Corona-Pandemie kein anderes Verhalten als Patienten ohne diese Sorgen. Möglicherweise überwogen die Sorgen vor Sehverlust gegenüber einer potenziellen Ansteckung mit dem Coronavirus, während die unterschiedlich stark ausgeprägten Beschwerden eines Myokardinfarktes nicht im gleichen Maße als ernst wahrgenommen und zunächst abgewartet wurden.

Zusammenfassend zeigten sich in dieser Arbeit im Vergleich zum Vorjahreszeitraum während der Corona-Pandemie erfreulicherweise keine signifikanten Unterschiede in der Schwere der Fälle oder der Versorgung von Patienten mit Amotio retinae. Unsere Ergebnisse geben damit Anhalt dafür, dass die Versorgung von ophthalmologischen Notfallpatienten während der Corona-Pandemie auf einem hohen Niveau aufrechterhalten werden konnte.

## Limitationen

Aufgrund der besonderen Situation während der Corona-Pandemie erfolgte eine retrospektive Untersuchung klinischer Routinedaten über einen zeitlich begrenzten Zeitraum. Es wurde eine begrenzte Studienpopulation eines variablen Krankheitsbildes untersucht. Eine Interpretation der Auswertung der nicht standardisierten Aufzeichnungen zur Anamnese sollte zurückhaltend erfolgen. Über die Ursache der im Vergleich zum Vorjahreszeitraum erhöhten Fallzahl lässt sich nur mutmaßen. Ein verändertes Verhalten von Zuweisern und umliegenden Kliniken ist eine mögliche Ursache und sollte in weiteren Arbeiten untersucht werden. Eine Untersuchung des postoperativen Verlaufs oder weiterer ophthalmologischer Notfälle erfolgte nicht.

## Ausblick

Die Ergebnisse dieser Arbeit geben Anhalt zu vermuten, dass sich die Versorgung von Patienten mit dem ophthalmologischen Notfall einer Netzhautablösung während der Corona-Pandemie nicht signifikant verschlechterte. Ob sich dies auch in einer Analyse des langfristigen funktionellen und anatomischen Erfolgs bestätigt, bleibt abzuwarten. Zur Bestätigung der vorliegenden Ergebnisse sollte eine multizentrische Arbeit mit größerer Studienpopulation auch das Notfallmanagement unterschiedlicher Kliniken beleuchten.

## Fazit für die Praxis

- Patienten mit Symptomen einer Netzhautablösung zögerten trotz der Corona-Pandemie nicht, den Augenarzt aufzusuchen, und die Schwere der behandelten Netzhautablösungen unterschied sich nicht signifikant zum Vorjahreszeitraum.
- Während der Corona-Pandemie konnte die Versorgung von Patienten mit Amotio retinae auf einem hohen Niveau aufrechterhalten werden.
