# Erratum zu: Amotio-retinae-Versorgung während der Corona-Pandemie: Suchten Patienten mit Netzhautablösung während der Corona-Pandemie später den Augenarzt auf? Ergebnisse aus einer deutschen Universitätsaugenklinik

**DOI:** 10.1007/s00347-021-01453-x

**Published:** 2021-07-12

**Authors:** N. Kaupke, M. S. Spitzer, R. Kromer

**Affiliations:** Klinik und Poliklinik für Augenheilkunde, Universitätsklinikum Hamburg-Eppendorf (UKE), Martinistr. 52, 20246 Hamburg, Deutschland

**Erratum zu:**

**Ophthalmologe 2020**

10.1007/s00347-020-01248-6

Der Artikel „Amotio-retinae-Versorgung während der Corona-Pandemie“ von Kaupke, N., Spitzer, M. S. und Kromer, R. wurde ursprünglich am 10. Oktober 2020 ohne „Open Access“ online auf der Internetplattform des Verlags publiziert. Die Autoren haben sich jedoch nachträglich für eine „Open Access“-Veröffentlichung entschieden. Das Urheberrecht des Artikels wurde deshalb am 12. Juni 2021 in © Der/die Autor(en) 2020 geändert. Der Artikel wird nun unter der Creative Commons Namensnennung 4.0 International Lizenz veröffentlicht, welche die Nutzung, Vervielfältigung, Bearbeitung, Verbreitung und Wiedergabe in jeglichem Medium und Format erlaubt, sofern Sie den/die ursprünglichen Autor(en) und die Quelle ordnungsgemäß nennen, einen Link zur Creative Commons Lizenz beifügen und angeben, ob Änderungen vorgenommen wurden.

Die in diesem Artikel enthaltenen Bilder und sonstiges Drittmaterial unterliegen ebenfalls der genannten Creative Commons Lizenz, sofern sich aus der Abbildungslegende nichts anderes ergibt. Sofern das betreffende Material nicht unter der genannten Creative Commons Lizenz steht und die betreffende Handlung nicht nach gesetzlichen Vorschriften erlaubt ist, ist für die oben aufgeführten Weiterverwendungen des Materials die Einwilligung des jeweiligen Rechteinhabers einzuholen. Weitere Details zur Lizenz entnehmen Sie bitte der Lizenzinformation auf http://creativecommons.org/licenses/by/4.0/deed.de.

